# Landscape‐dependent effects of varietal mixtures on insect pest control and implications for farmer profits

**DOI:** 10.1002/eap.2246

**Published:** 2021-01-06

**Authors:** Lauren D. Snyder, Miguel I. Gómez, Erika L. Mudrak, Alison G. Power

**Affiliations:** ^1^ Department of Ecology and Evolutionary Biology Cornell University Ithaca New York 14853 USA; ^2^ Charles H. Dyson School of Applied Economics and Management Cornell University Ithaca New York 14853 USA; ^3^ Cornell Statistical Consulting Unit Cornell University Ithaca New York 14853 USA

**Keywords:** *Brassica oleracea*, ecosystem services, insect pests, intraspecific trait variation, farmer profits, landscape composition, varietal mixtures

## Abstract

Intraspecific plant diversity can significantly impact insect herbivore populations in natural systems. Yet, its role as an insect pest control strategy in agriculture has received less attention, and little is known about which crop traits are important to herbivores in different landscape contexts. Moreover, empirical economic analyses on the cost‐effectiveness of varietal mixtures are lacking. We used varietal mixtures of *Brassica oleracea* crops on working farms to examine how two metrics of intraspecific crop diversity, varietal richness and number of plant colors (color richness), affect crop damage and the incidence and abundance of two insect pest species: *Pieris rapae* and *Phyllotreta* spp. We evaluated the context‐dependency of varietal mixtures by sampling early‐ and late‐season plantings of *B. oleracea* crops in farms across a gradient of landscape composition. We developed crop budgets and used a net present value analysis to assess the impact of varietal mixtures on input and labor costs, crop revenues, and profit. We found context‐dependent effects of varietal mixtures on both pests. In early‐season plantings, color richness did not affect *Phyllotreta* spp. populations. However, increasing varietal richness reduced *Phyllotreta* spp. incidence in simple landscapes dominated by cropland, but this trend was reversed in complex landscapes dominated by natural habitats. In late‐season plantings, color richness reduced the incidence and abundance of *P. rapae* larvae, but only in complex landscapes where their populations were highest. Varietal richness had the same effect on *P. rapae* larvae as color richness. Unexpectedly, we consistently found lower pest pressure and reduced crop damage in simple landscapes. Although varietal mixtures did not affect crop damage, increasing color richness corresponded with increased profits, due to increased revenue and a marginal reduction in labor and input costs. We demonstrate varietal mixtures can significantly impact pest populations, and this effect can be mediated by intraspecific variation in crop color. However, the strength and direction of these effects vary by season, landscape composition, and pest species. The association between varietal color richness and profitability indicates farmers could design mixtures to enhance economic returns. We recommend additional research on the benefits of intraspecific trait variation for farmers.

## Introduction

Low‐diversity intensified agricultural systems have been implemented on a global scale in an effort to produce sufficient food for a growing human population. Although these systems can be highly productive when supplied with external inputs, valuable ecosystem services such as pest control are often not supported (Altieri 1999, Power 2010). Increasing crop species diversity in the form of polycultures is an agroecological strategy long used by smallholder farmers around the world, and a strong body of literature shows polycultures support pest control services (Andow 1991, Letourneau et al. 2011). This service may be driven by natural enemies or by variation in crop traits, such as phytochemistry, color, or morphology (Cartea et al. 2010), which can diminish herbivores' ability to locate preferred host plants, as suggested by the resource concentration hypothesis (Root 1973). However, despite potential pest control services and other benefits, polycultures can be costly. For instance, polycultures may not lend themselves to mechanized agricultural equipment, thereby increasing labor requirements, and they demand more agronomic knowledge than monocultures because growers must be familiar with the planting times, management needs, and marketability of each crop species (Gliessman 1985).

A growing body of research suggests that intraspecific plant diversity, genetic diversity within a plant species, may be as important in structuring insect communities as plant species diversity (Tooker and Frank 2012, Crawford and Rudgers 2013). For some farmers, increasing crop diversity in the form of varietal mixtures may be a more feasible management practice than polycultures because they require relatively minor changes to agricultural practices (Tooker and Frank 2012, Casagrande et al. 2017). For instance, farmers could expand varietal diversity in a crop species they already grow, thus providing a less costly diversification strategy. The potential for varietal mixtures to provide a less labor‐intensive diversification strategy is particularly appealing, because labor is often the most expensive and difficult input for growers to procure (Bronars 2014, Duvall 2017). However, to our knowledge, there are no empirical studies evaluating the impact of varietal mixtures on labor costs and grower profits, which impedes our ability to provide *effective* recommendations to growers.

While many studies have examined the effect of varietal mixtures on crop yield (Reiss and Drinkwater 2018) and pathogen prevalence (Mundt 2002), fewer studies have explored the effect of varietal mixtures on insect herbivores in agricultural systems (reviewed in Tooker and Frank 2012). Furthermore, most studies of insect herbivore dynamics in varietal mixtures have focused on whether varietal mixtures influence insect population densities, and there has been less emphasis on understanding which crop traits are responsible (but see Wetzel et al. 2016, 2018). Studies from natural systems indicate that insect herbivores respond to intraspecific variation in morphological plant traits, such as leaf color, in genotypic mixtures (Sinkkonen et al. 2012, Green et al. 2015). However, the effect of intraspecific trait variation in crop varietal mixtures on agricultural insect pests is poorly understood.

Some studies in agricultural systems have shown a strong effect of varietal mixtures on insect populations (Altieri and Schmidt 1987, Power 1988), while others have shown little or no effect (Cantelo and Sanford 1984, Power 1988). We suggest the mixed results from these studies could be related to spatial and temporal variation in the dynamics of insect pests and their natural enemies. For example, landscape composition is known to strongly influence insect populations (Bianchi et al. 2006, Tscharntke et al. 2007) and the effectiveness of management practices at the local‐scale (Tscharntke et al. 2012). Landscapes with a high proportion of natural areas are frequently associated with high natural enemy abundance, which in turn can enhance pest control services (Gardiner et al. 2009, Chaplin‐Kramer et al. 2011). Measuring temporal dynamics is also important because pest and natural enemy populations, along with crop damage, fluctuate over a growing season (Chaplin‐Kramer et al. 2013). For example, in brassica crops grown in central New York State, flea beetles (*Phyllotreta* spp.) are the primary insect pests in the early growing season (April–June), while the small white butterfly (*Pieris rapae*) becomes a dominant pest in the late growing season (June–August; Seaman 2013). Therefore, the effectiveness of varietal mixtures as a pest management strategy is likely to vary across time and space. While previous field studies on varietal mixtures have accounted for temporal variation by sampling pests throughout a growing season, little is known about the interaction between varietal mixtures and landscape composition.

Here, we evaluate the effects of early‐and late‐season plantings of *Brassica oleracea* mixtures (local‐scale intraspecific crop diversity) and the percent of cropland at the landscape‐scale on the incidence and abundance of two important cruciferous pests, *P. rapae* and flea beetles, and resulting crop damage. In addition, we explore the role of plant color in mediating pest response. While *B. oleracea* varieties vary in a number of functional traits, such as plant structure and phytochemical composition, in this study, we focus on intraspecific variation in plant color for several reasons: (1) cruciferous herbivores are known to use color as a visual cue in host selection (Yang et al. 2003, Tsuji and Coe 2014), (2) color is correlated with other functional traits, including crop chemical profiles (Choi et al. 2014), which are also known to be important host identification cues for cruciferous pests (Nielsen 1988, Renwick and Radke 1988), and (3) color can be measured easily in the field and would be straightforward for farmers to manipulate. Finally, we evaluate the potential for varietal mixtures to serve as a profitable form of crop diversification by measuring their effect on labor costs and profitability. We predict that (1) the effect of varietal mixtures on pest incidence/abundance and crop damage will depend on landscape context and time of growing season (e.g., early vs. late season), (2) varietal color richness will mediate the effect of mixtures on pests, and (3) increasing intraspecific crop diversity will increase grower profits.

We investigated these predictions by measuring insect pest incidence and abundance, crop damage, and grower profitability in farmer fields located across a gradient of cropland cover in upstate New York during early and late‐season *B. oleracea* plantings.

## Methods

### Study system

We sampled early‐ and late‐season insect pests on crucifers in the Finger Lakes region of New York State on farms growing the following *B. oleracea* varieties: broccoli, brussels sprouts, cabbage, cauliflower, collards, kale, and kohlrabi. *Brassica oleracea* contains a large amount of intraspecific variation (Ahuja et al. 2010), which increased our likelihood of detecting an effect of varietal mixtures and made it possible to focus on a conspicuous crop trait, plant color, that could be mediating herbivore abundance. While *B. oleracea* contains more morphological variation compared to many other crop species, *B. oleracea* varieties have similar agronomic requirements and therefore have the potential to reduce some of the logistical complications associated with polycultures. For example, *B. oleracea* varieties have very similar climatic and soil fertility requirements (Guerena 2006). Moreover, because *B. oleracea* varieties share similar pest complexes (Seaman 2016), a farmer’s knowledge of problem pests for one variety would be broadly applicable to other varieties. In our study region, most *B. oleracea* varieties are transplanted rather than direct seeded (Seaman 2016), and multiple varieties could be transplanted as a mixture using a single mechanized transplanter.

We focused on two important pests of crucifers: the foliar feeding lepidopteran *P. rapae* and flea beetles (*Phyllotreta cruciferae* and *Phyllotreta striolata*; Ahuja et al. 2010). In our study region, crucifers are planted multiple times from April through August and harvested from July through November (Seaman 2013). Flea beetles are dominant early in the season and their populations begin to decline in June, while *P. rapae* populations peak later in the season, typically after June (Seaman 2013). To address these seasonal differences, we focused our sampling on the late‐season planting in 2013 to capture *P. rapae* dynamics, and the early‐season planting in 2014 to capture flea beetle dynamics.

### Survey design

We sampled insect pests on farms growing *B. oleracea* varieties in fields with low (one variety) to high (six varieties) numbers of varieties (i.e., varietal richness). We also recorded the number of varietal colors (color richness) in each field. Varietal richness and color richness often vary independently in these mixtures. For example, farmers may plant a purple sub‐variety of cauliflower, a white sub‐variety of cauliflower, and a green sub‐variety of broccoli in the same field. In this scenario, we would record two varieties (cauliflower and broccoli), but three colors (purple, white, and green).

We monitored pest incidence and abundance at 19 fields on 14 farms in the early season and 23 fields on nine farms in the late season, which included all farms from the early‐season survey. Farms were separated by a minimum of 3.5 km in the early season and 4 km in the late season. Separation between fields associated with the same farm ranged from 40 m to 4680 m in the early season (mean = 870.7 m, median = 119 m) and 29 m to 2000 m in the late season (mean = 285.4 m, median = 150 m). The fields ranged in size from 21 to 26,246 m^2^. Although agricultural management varied by farm, all farms were characterized by organic practices; not all farms were certified organic, but all followed organic production practices and no synthetic inputs were used (Appendix S1: Table S1). Given the observational nature of our study, planting schemes varied across farms, but all varieties present in mixtures were planted in close proximity. Different varieties were either planted together within a row or planted next to one another in alternating rows. We did not observe varieties arranged in large blocks or strips.

We used the proportion of cropland as our landscape composition metric, a land cover type influential for flea beetles (Andersen et al. 2005) and *P. rapae* (Benson et al. 2003). Crops in this region included vegetables, fruits, legumes, cereals, and fallow fields. We selected fields to represent a gradient of landscape complexity, ranging from simple landscapes composed mostly of cropland (71% cropland) to complex landscapes with little cropland (5% cropland). We define landscapes with little cropland as complex because non‐agricultural land in our study region is comprised of a variety of natural areas, including deciduous, evergreen, and mixed forests, wetlands, and pastures composed of clover, wildflowers, and grasses. For each field, percent cropland at three spatial scales (500, 1,000, and 1,500 m radius) was calculated using the 2013 and 2014 Crop Data Layers (USDA National Agricultural Statistics Service Cropland Data Layer), respectively, and ArcGIS software (ArcMap version 10.1, ESRI, Redlands, California, USA).

### Pest sampling methods

We captured early‐season pest dynamics by visually counting adult flea beetles on 30 plants in each field three times from May to early July. At each sample event, we selected six random points in each field. At each point, we visually inspected five consecutive plants for adult flea beetles. Each plant was assessed for pest damage using a damage index ranging from zero (no damage) to five (severe damage) based on methods from Macharia et al. (2005) (Appendix S1: Table S2). To account for seasonal changes in pest incidence/abundance and crop damage, we partitioned our sampling schedule into three two‐week sampling periods: mid‐May, early June, and late June to early July. Due to differences in farmers’ planting and harvesting schedules, fields were sampled during at least two of the sampling periods, but never more than once per sampling period.

We measured the incidence and abundance of *P. rapae* in the late season by counting the number of larvae on 30 plants in each field from July through early September following the same random sampling scheme used in the early‐season survey. Each plant was assessed for pest damage using the same damage index described for the early‐season sampling. Based on planting and harvesting schedules, we partitioned our sampling schedule into five sampling periods two weeks in length: early July, late July, early August, late August, and early September. All fields were sampled one to five times over the growing season, but never more than once per sampling period.

### Crop budgets

We developed crop budgets for 10 of the 19 farm fields sampled in the 2014 early‐season survey using a net present value (NPV) approach to calculate the cost of inputs, revenue generated by yield, and profitability (methods based on McCarl 1982, Wiswall 2009, Atallah and Gómez 2013). The crop budgets provide the basis of the economic comparison between low‐ and high‐diversity fields. NPV analysis calculates the difference between the present value of cash inflows and the present value of cash outflows, which enabled us to analyze the profitability of increasing varietal and color richness. We defined profitability as the total revenue generated by yield minus total expenses, and categorized expenses by the type of cost. We explicitly evaluated the effect of intraspecific crop diversity on labor because it is one of the most limiting inputs for growers (McCarl 1982, Bronars 2014). Therefore, we separated expenses into two categories: input costs (transplant, pest management, weed management, and fertility costs) and labor costs. When possible, financial information was gathered directly from farmers’ records. Incomplete farm records were augmented with the average input and output prices used by the other farms in the study.

### Statistical methods

For all analyses, we developed hypothesis‐based linear models in R Statistical Software 3.4.1 (R Core Team 2017) to test our predictions that both local‐ and landscape‐scale diversity would impact pest incidence/abundance and crop damage. We used variance inflation factors (VIF) to ensure that the explanatory variables included in our models were not collinear (Zuur et al. 2009). Based on methods in Zuur et al. (2009), we used a VIF cut‐off value of three to define collinear variables; all explanatory variables included in our models were below this cut‐off value, indicating that none of our predictors were collinear.

Each of the models discussed below were fitted separately using the percent cropland variable calculated at each of three spatial scales (500, 1,000, and 1,500 m) and Akaike Information Criterion (AIC) values were used to determine the most predictive scale (Burnham and Anderson 2002). Because we focused on late‐season pest dynamics in 2013 and early‐season dynamics in 2014, we analyzed each year separately. To facilitate interpretation, all figures were plotted on the transformed scale, but labeled with untransformed values. We used Mantel tests to check for spatial autocorrelation of landscape composition at farm fields, as well as for spatial autocorrelation between the fields and response variables. Mantel tests indicate our data were not spatially autocorrelated either year (see Appendix S1: Tables S3 and S4).

#### Pest dynamics

To analyze pest incidence and abundance we used generalized linear mixed effects models fitted with the glmer function in the lme4 package (Bates et al. 2015). Explanatory variables included varietal richness, color richness, percent cropland, and two‐way interactions between percent cropland and the local‐scale predictors: varietal richness and color richness. We used a nested random effects design of sampling point nested within sampling period nested within field. By including field as a random effect, we accounted for variation in field size, planting scheme, and management style.

To accommodate zero‐inflated and overdispersed count data, we employed manual two‐step hurdle models (Zuur et al. 2009). This amounted to two separate models: one for the binary “presence” or “absence” of a pest (incidence), and the second for the number of individuals present (abundance). In the first step, we modeled pest incidence using a binomial distribution (logit link) to govern the binary outcome of whether a pest existed on that plant. In the second step, we modeled pest abundance when present (i.e., the non‐zero outcomes) with a Poisson distribution (log link). To check for overdispersion in the Poisson models, we used the overdisp.glmer function in the RVAideMemoire package (Herve 2017). If the model was overdispersed, an observation level random effect was added to correct for overdispersion (Harrison 2014). To ensure our data were well‐modeled by the specified distributions as well as to check for violations of homoscedasticity and linearity, we used the DHARMa package (Hartig 2017) in R; no anomalies were found. The slopes of fitted lines were estimated and compared using the lstrends function in the lsmeans package (Lenth 2016).

#### Crop damage

To evaluate how crop damage was affected by varietal richness, color richness, percent cropland, and the two‐way interactions between percent cropland and the local‐scale predictors, we fit linear mixed effects models using the lmer function (lme4 package; Bates et al. 2015). We analyzed the relationship between crop damage and pest incidence/abundance using linear mixed effects models. Again, our random effects included sampling point nested within period nested within field. The effects of fixed factors and interaction terms were evaluated using Type II Wald χ^2^ tests with the Anova function in the car package (Fox and Weisberg 2011).

#### Crop budgets

In addition to exploring the effect of varietal mixtures on overall field profitability, we were interested in their relationship with production costs (i.e., labor and input costs) and revenue generation. Thus, the crop budget data set included response variables of profitability, labor costs, input costs, and revenue generated from crop yield. Given that labor costs, input costs, and revenue were variables used to calculate profitability, it is likely these variables are not independent of one another. To avoid a Type I error, we used a Holm’s Procedure for multiple test correction. Corrected and uncorrected *P* values are reported in the supplementary materials; we used corrected *P* values to interpret our results. We lacked sufficient sample size (*n* = 10 fields) to investigate the impact of landscape predictors on the economic response variables. Therefore, we included only local‐scale predictors (i.e., varietal and color richness). Due to the small sample size, fields in the crop budget data set only had one or two varietal colors.

## Results

### Pest dynamics

**Fig. 1:**
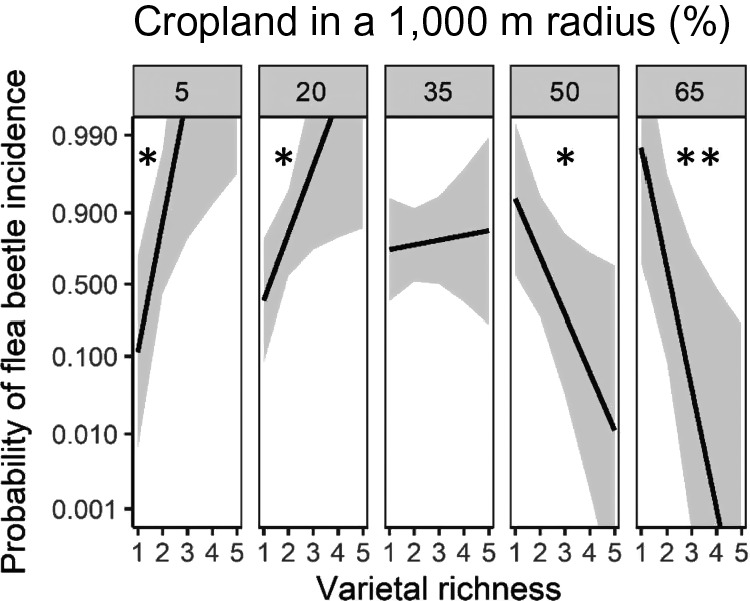
Model estimates with 95% confidence intervals for the relationship between flea beetle incidence and varietal richness across a gradient of landscape complexity (5%, 20%, 35%, 50%, and 65% cropland in a 1,000 m radius) in the early‐season planting. We show percent crop land at equal intervals that portray the range of landscape complexity in our study region and are well represented in our data set. Slope significantly different from one is indicated by *P < 0.05, **P < 0.01. The figure is plotted on the logit scale as it was modeled, but labeled with back‐transformed proportion values.

**Fig. 2:**
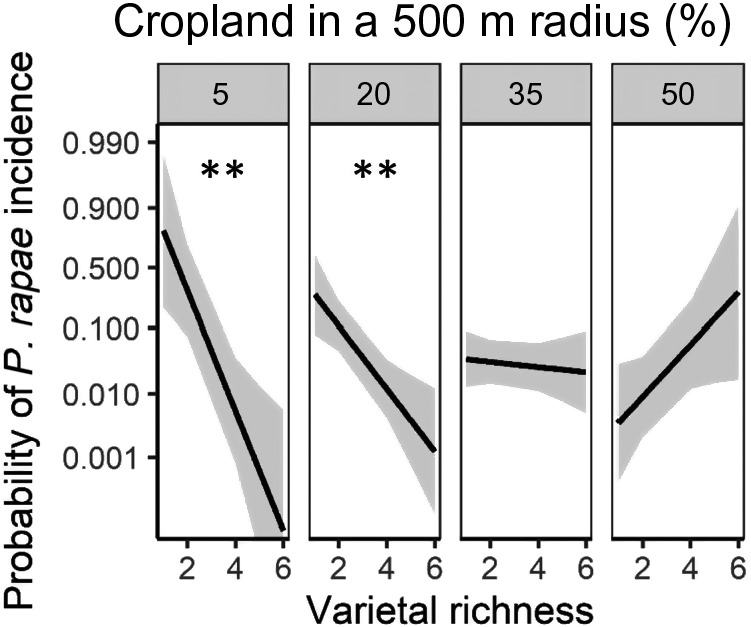
Modeled effect of varietal richness on *Pieris rapae* incidence across a gradient of landscape complexity (5%, 20%, 35%, and 50% cropland in a 500 m radius) in the late‐season planting. We show percent crop land at equal intervals that portray the range of landscape complexity in our study region and are well represented in our data set. The figure shows regression slopes with 95% confidence intervals. Slope significantly different from one is indicated by **P < 0.01. The figure is plotted on the logit scale as it was modeled, but labeled with back‐transformed proportion values.

**Fig. 3:**
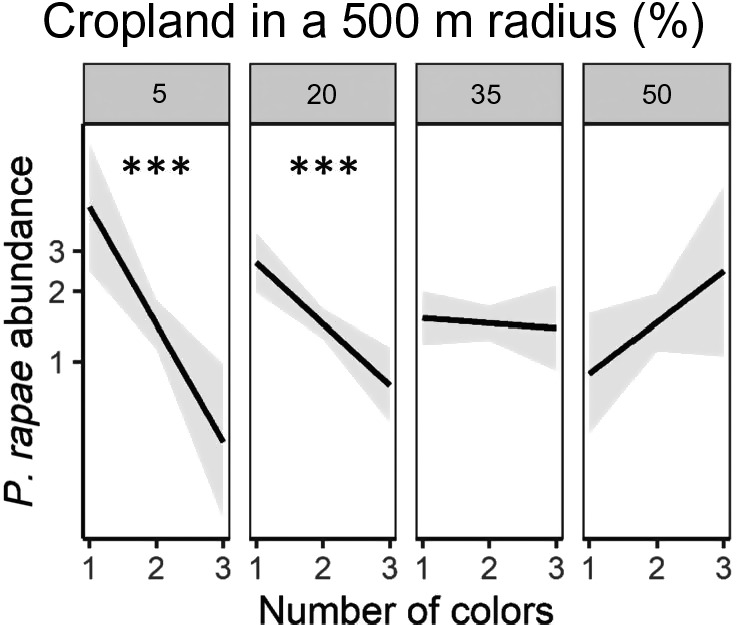
Modeled effect of the number of colors in a field on *P. rapae* abundance across a gradient of landscape complexity (5%, 20%, 35%, and 50% cropland in a 500 m radius) in the early‐season planting. We show percent crop land at equal intervals that portray the range of landscape complexity in our study region and are well represented in our data set. The figure shows regression slopes with 95% confidence intervals. Slope significantly different from one is indicated by ***P < 0.001

The effect of varietal and color richness on pest incidence and abundance varied across the landscape and by pest. We show results at the 1,000‐ and 500‐m scales for the early‐season and late‐season surveys, respectively, because these were the most predictive scales for the dynamics of the focal pest (Appendix S1: Table S5). In the early season, we found a significant interaction between landscape complexity and varietal richness (*P* = 0.007; Appendix S1: Table S5). There was a negative association between flea beetle incidence and varietal richness in simple landscapes (≥50% cropland; *P* < 0.05; Fig. 1). However, this trend was reversed in complex landscapes (≤20% cropland; *P* < 0.05; Fig. 1). We found no effect of color richness on flea beetles. We also found a landscape‐dependent effect of varietal richness on *P. rapae* incidence in the late season (*P* < 0.004; Appendix S1: Table S5). That is, increased varietal richness had a significant negative effect on *P. rapae* incidence, but only in complex landscapes (≤20% cropland; *P* < 0.01; Fig. 2;). Similarly, as color richness increased, *P. rapae* abundance significantly decreased, but only in complex landscapes (*P* < 0.001; Fig. 3; Appendix S1: Table S5). Furthermore, as the proportion of cropland in the landscape increased, the abundance of *P. rapae* larvae significantly decreased, indicating that *P. rapae* was less abundant in simple landscapes (*P* < 0.05; Appendix S1: Table S5). Our results indicate that flea beetles and *P. rapae* had unique responses to the interaction between intraspecific *B. oleracea* diversity and landscape composition.

### Crop damage

**Fig. 4:**
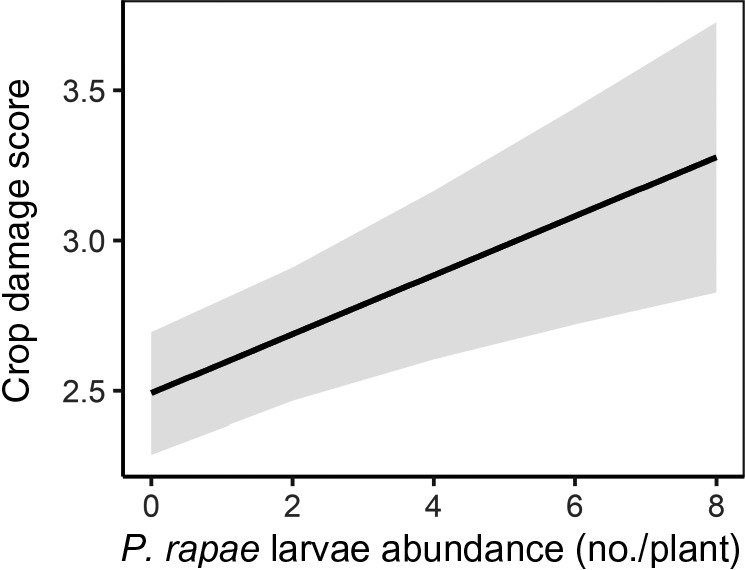
Model estimates with 95% confidence intervals for the relationship between crop damage and *Pieris rapae* larvae abundance (predicted mean number of *P. rapae* larvae per plant) in the late‐season planting.

**Fig. 5:**
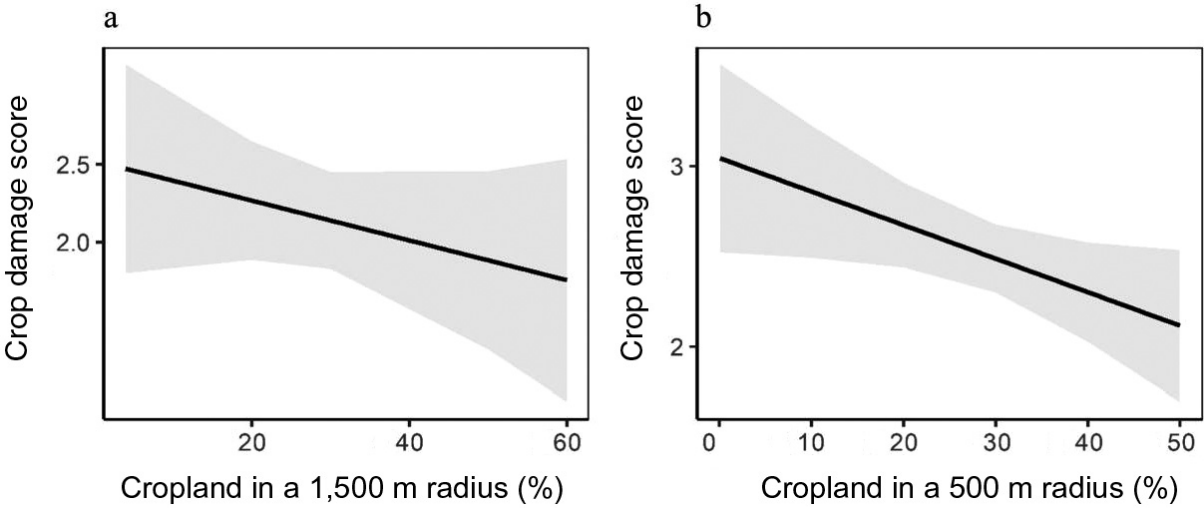
(a) Model estimates with 95% confidence intervals for the relationship between crop damage and percent cropland at the 1,500‐m scale in the early‐season planting. (b) Model estimates with 95% confidence intervals for the relationship between crop damage and percent cropland at the 500‐m scale in the late‐season planting.

Flea beetles had no effect on crop damage in the early season, but crop damage was significantly associated with *P. rapae* abundance in the late season (*P* < 0.001; Fig. 4). Varietal richness and color richness were not significantly correlated with crop damage in either the early‐or late‐season survey. However, throughout the growing season, crop damage decreased significantly as the percent of cropland in the surrounding landscape increased (*P* < 0.05; Fig. 5). This effect was significant at the 1,000‐ and 1,500‐m scale in the early‐season survey and at the 500‐m and 1,000‐m scale in the late‐season survey.

### Crop budgets

**Fig. 6:**
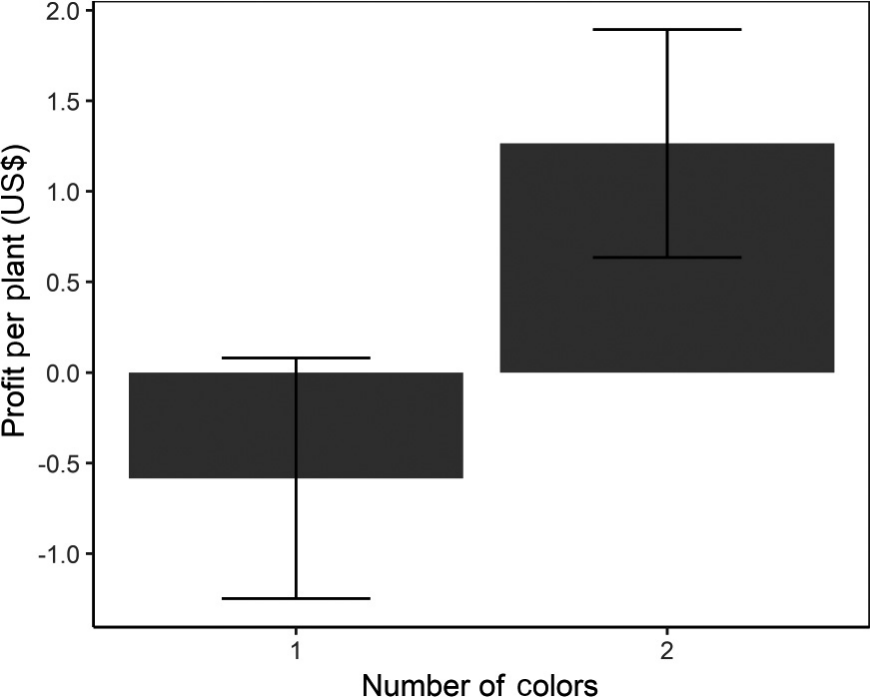
Effect of color richness on profitability. The bars represent the model mean estimate and the error bars represent standard error.

Fields with two varietal colors had significantly higher profits than fields with only one varietal color (*P* < 0.05; Fig. 6; Appendix S1: Table S6). Moreover, increasing the number of colors in a field was associated with a significant increase in revenue (*P* < 0.01; Appendix S1: Table S6), and a marginally significant reduction in labor costs (*P* = 0.066; Appendix S1: Table S6) and input costs (*P* = 0.068; Appendix S1: Table S6). We found no significant effect of varietal richness on profitability, revenue, labor costs, or input costs.

## Discussion

Our findings highlight the importance of accounting for spatial and temporal variation while evaluating the efficacy of local management practices, as the effect of varietal mixtures on pest populations varied across landscapes and growing season. The variability we detected may partially explain the previously documented mixed effects of varietal crop mixtures on insect pests (e.g., Cantelo and Sanford 1984, Altieri and Schmidt 1987, Power 1988, 1991). Moreover, we found that intraspecific variation in crop color can significantly influence pest populations and profitability, suggesting that using a trait‐based approach to design varietal mixtures could maximize the value of the ecosystem services provided.

In simple landscapes, the incidence of flea beetles decreased significantly in response to intraspecific crop diversity. One explanation for this trend could be related to higher variability in plant chemistry in varietal mixtures. There is growing evidence that increased variability in plant chemistry can elicit energetically costly physiological responses in insect herbivores, constraining their population size (Wetzel and Thaler 2016). For instance, a recent study on Japanese beetles (*Popillia japonica*) revealed that beetles foraging across a suite of host plant species induce detoxification enzymes allowing them to cope with a greater diversity of plant chemical defenses (Adesanya et al. 2016); however, this detoxification process incurs a high energetic cost (Karban and Agrawal 2002). Varieties of *B. oleracea* are known to vary widely in their phytochemical profiles, including compounds important to plant defense against herbivores (Ahuja et al. 2010). Fields with high *B. oleracea* diversity may present a greater diversity of phytochemicals, thus requiring flea beetles to increase production of detoxification enzymes. Furthermore, some insect herbivores exhibit a similar detoxification response when exposed to pesticides (Schuler 2011), and landscapes dominated by agriculture have been shown to have higher background levels of pesticides compared to landscapes with a high proportion of seminatural habitats (Meehan et al. 2011). Flea beetles foraging in fields with high *B. oleracea* diversity surrounded by agriculture may experience relatively high enzymatic costs. An inability to detoxify an array of phytochemicals as well as pesticides may partially explain the negative relationship between intraspecific *B. oleracea* diversity and flea beetle incidence in simple landscapes.

In complex landscapes, intraspecific *B. oleracea* diversity had a positive effect on flea beetle incidence. While this result contrasts with other studies in agricultural systems showing a negative relationship between intraspecific crop diversity and herbivore abundance (Koricheva and Hayes 2018), it is consistent with multiple studies in natural systems demonstrating a positive association between intraspecific plant diversity and herbivore abundance (Crutsinger et al. 2006, McArt and Thaler 2013). Fields with high intraspecific diversity may provide a greater diversity of dietary resources, as suggested by the balanced diet hypothesis (Tilman 1982, DeMott 1998). Therefore, in complex landscapes where herbivores may be less exposed to pesticides, it is possible that the nutritional benefits associated with dietary diversity outweigh the enzymatic costs associated with increased plant phytochemical diversity.

We did not explicitly explore the role of phytochemicals in our study, as our primary goal was to investigate a crop trait (plant color) that is simple to categorize and that farmers could easily manipulate. Nonetheless, we recognize that crop chemical profiles can be correlated with plant color and may also influence plant host selection by insect pests. For example, a study assessing the chemical profiles of white, yellow, green, and purple cauliflower varieties found strong correlations between varietal color and plant chemistry (Park et al. 2013). Specifically, anthocyanins were only detected in purple cauliflower varieties, while green cauliflower varieties had considerably higher levels of carotenoids compared to the other varieties (Park et al. 2013). These plant chemicals are known to play important roles in plant defense against insect pests. The optical properties of anthocyanins can serve as visual signals to insect herbivores, potentially communicating a strong investment by the plant in chemical defenses that are toxic or unpalatable (Lev‐Yadun and Gould 2009). Carotenoids not only play a role in plant visual cues to insect pests, they are also important for plant chemical defense pathways that are induced in response to herbivory (Heath et al. 2013). However, crop chemical profiles also vary significantly by plant age and morphology and abiotic conditions (Choi et al. 2014). Previous studies examining chemical profiles of red cabbage genotypes found significant variation in anthocyanin content and profiles across the genotypes (Wiczkowski et al. 2014, Strauch et al. 2019). Indeed, the concentration of some anthocyanins can be almost five times greater in one red cabbage genotype compared to another (Wiczkowski et al. 2014). Therefore, it is unlikely that there is a straightforward relationship of one varietal color to one chemical profile.

We conducted our study on working farms, and the specific varieties and colors varied across farms; therefore, we were unable to evaluate whether there is a correlation between varietal color and plant chemistry using our data set. However, the strong relationship between color richness and herbivore abundance established in our study suggests that color is a useful and logistically feasible management strategy for farmers to implement. This finding should motivate future research exploring whether color itself drives the observed effect on herbivores or if there are underlying chemical profiles associated with color that contribute to the color effects observed in this study.

In contrast to its effect on flea beetle dynamics, varietal richness reduced the incidence of *P. rapae* in complex landscapes, but had no effect in simple landscapes. A recent quantitative synthesis of the effect of landscape composition on insect pest species revealed substantial variation in pest species’ responses to landscape variables (Karp et al. 2018). Our results concur with these findings, as we showed that flea beetles and *P. rapae* exhibit contrasting responses to landscape composition. In New York State, *P. rapae* is a host for several parasitoid species (Shelton et al. 2002), which have been shown to cause high levels of mortality in early instars of *P. rapae* (Herlihy et al. 2012). Non‐crop habitats such as forests and wetlands are important sources of refuge and alternative foods for natural enemies (Landis et al. 2000), and thus their populations are often higher in complex landscapes (reviewed in Chaplin‐Kramer et al., 2011). Research in natural systems has demonstrated that intraspecific plant diversity supports a higher abundance and diversity of parasitoids (Jones et al. 2011). The suppressive effect of intraspecific crop diversity that we observed in complex landscapes could be a result of enhanced biocontrol services. In our study, *P. rapae* was significantly less abundant in simple landscapes compared to complex landscapes. This finding appears to be in conflict with the resource concentration hypothesis, which would suggest that herbivore abundance should be highest in areas with concentrated food resources (Root 1973). Higher background levels of pesticides, often associated with simplified landscapes (Meehan et al. 2011), may have reduced the overall abundance of *P. rapae* in these areas. Indeed, previous work suggests that the effect of pesticides can outweigh effects of landscape composition on pest abundance (Veres et al. 2013). The low density of *P. rapae* that we observed in simple landscapes may have hindered detection of an effect of varietal mixtures in these landscapes.

Our results suggest that varietal color is one crop trait that has the potential to influence some pest species. Color richness elicited the same response from *P. rapae* as varietal richness; in complex landscapes, increased color richness reduced the abundance of *P. rapae* larvae. Since varietal and color richness vary independently from one another and are not collinear, these results demonstrate that *P. rapae* responded to intraspecific variation in crop color in addition to overall varietal richness. Previous studies have demonstrated that color is an important plant trait in moderating insect populations and *P. rapae* in particular is known to use foliage color as a landing cue (Tsuji and Coe 2014). In some instances, plant color is also correlated with plant defense compounds (Malenčić et al. 2012, Green et al. 2015). Therefore, it is possible that *P. rapae* responded to chemical as well as visual cues associated with color richness. In contrast, flea beetles did not respond to color richness, indicating that other plant traits play a more important role in mediating their population dynamics. *Brassica oleracea* varieties also differ widely in plant structure and chemistry (Ahuja et al. 2010), and previous research has demonstrated that an herbivore’s response to structural and biochemical traits in *B. oleracea* crops can be species specific (Santolamazza‐Carbone et al. 2013). Future studies are needed to further tease apart the varietal traits underlying the effect of mixtures on different insect species.

Although intraspecific crop diversity significantly influenced pest populations under certain scenarios, it had no effect on crop damage in either early‐ or late‐season plantings. Rather, crop damage was negatively correlated with the percentage of cropland in the landscape throughout the growing season, consistent with the negative relationship between *P. rapae* abundance and percent cropland in the late season. In our study, lower crop damage in simple landscapes may be a result of reduced pest pressure. Although our early‐season sampling occurred before *P. rapae* reached peak abundance, it is possible that the effect of landscape complexity on crop damage in the early‐season planting was also driven by *P. rapae*, as its abundance would have been increasing towards the end of our sampling period (Seaman 2013).

Although we did not find an effect of varietal mixtures on crop damage in this study, one limitation was the unbalanced design of the varietal mixtures we sampled. Because we conducted our research on working farms, the particular varieties that were included in the mixtures varied from field to field. Given the importance of plant trait diversity in moderating ecosystem services (Wood et al. 2015), the potential for crop varietal mixtures to support pest control services could likely be increased by deliberately designing mixtures to include specific varietal traits.

Our economic analysis suggests that varietal mixtures warrant further attention due to their potential to provide economic benefits. Despite a relatively small sample size, our results demonstrate that varietal mixtures can enhance profitability, as we found that increasing color richness significantly increased profit. The effect of color richness on profit appears to be mediated via its effect on revenue, and potentially on labor and input costs, as well; we found that increased color richness was associated with a significant increase in revenue generated from yield, and a marginally significant reduction in labor and input costs. We suggest that the effect of color richness on revenue could be a result of enhanced pest control services. In complex landscapes, the abundance of *P. rapae* was reduced in plots with high color richness, and *P. rapae* was associated with crop damage. Therefore, increasing color richness may have increased marketable yield without increasing, and potentially even decreasing, the amount of labor and inputs a grower had to allocate to pest management via a reduction in *P. rapae* abundance and crop damage. The fact that our economic analysis reveals an effect of color richness, but not varietal richness, is further indication that intraspecific variation in specific crop traits, rather than simply varietal diversity, drives ecological and economic services.

While the conclusions drawn from the economic analysis could be tempered by the limited sample size, our findings suggest that varietal mixtures of *B. oleracea* could serve as a profitable management strategy and should motivate further research into their ability to mitigate implementation constraints associated with other diversification strategies. To better understand the potential for *B. oleracea* mixtures to reduce implementation constraints, future studies should systematically evaluate the similarity of *B. oleracea* varieties across a spectrum of management categories, including growing requirements, planting and harvesting techniques, and marketing outlets. The finding that intraspecific crop diversity did not increase labor costs is particularly encouraging, as labor is often the most difficult input for growers to procure, particularly in the current U.S. context where the lack of a stable agricultural workforce has had substantial impacts on the national economy (Duvall 2017). Indeed, the farmers included in our economic analysis allocated an average of 47% of their input costs to labor. A diversification practice that does not entail a trade‐off between yield services and labor expenditures could be quite valuable for growers. This finding underscores the importance of explicitly measuring the value of services as well as production costs to determine overall profitability, arguably the most relevant metric for agricultural producers.

Future studies should also evaluate consumers’ willingness to purchase new varieties, particularly varieties that differ in color, because this appears to be a varietal trait relevant to insect pest control. Currently, many supermarkets around the world only offer one or a few varieties of a given crop species (Lamers et al. 2016). Evaluating consumer preferences for varietal diversity would be useful, since increased demand for diversification could provide an additional incentive for growers to implement varietal mixtures. The widespread adoption of varietal mixtures would require the availability of a diverse seed supply as well as markets for these varieties. Finally, to maximize value to growers, we suggest that future research on the design of varietal mixtures consider economically important varietal attributes related to ease of cultivation, labor demands, and market value in addition to traits related to environmental adaptability and productivity. Such information would support the thoughtful design of varietal mixtures that maximize both ecological and economic services for growers.

## Supporting information

Appendix S1

## Data Availability

Data are available from the Dryad Digital Repository (Snyder et al., 2020): https://doi.org/10.5061/dryad.0gb5mkkzc.

## References

[eap2246-bib-0001] Adesanya, A. , N. Liu , and D. W. Held . 2016. Host suitability and diet mixing influence activities of detoxification enzymes in adult Japanese beetles. Journal of Insect Physiology 88:55–62. 26964493 10.1016/j.jinsphys.2016.03.002

[eap2246-bib-0002] Ahuja, I. , J. Rohloff , and A. M. Bones . 2010. Defence mechanisms of Brassicaceae: implications for plant‐insect interactions and potential for integrated pest management. A review. Agronomy for Sustainable Development 30:311–348.

[eap2246-bib-0003] Altieri, M. A. 1999. The ecological role of biodiversity in agroecosystems. Agriculture, Ecosystems & Environment 74:19–31.

[eap2246-bib-0004] Altieri, M. A. , and L. L. Schmidt . 1987. Mixing broccoli cultivars reduces cabbage aphid numbers, 41:24–26. California Agriculture.

[eap2246-bib-0005] Andersen, C. L. , R. Hazzard , and R. V. A. N. Driesche . 2005. Overwintering and seasonal patterns of feeding and reproduction in *Phyllotreta cruciferae* (Coleoptera: Chrysomelidae) in the Northeastern United States. Environmental Entomology 34:794–800.

[eap2246-bib-0006] Andow, D. 1991. Vegetational diversity and arthropod population response. Annual Review of Entomology 36:561–566.

[eap2246-bib-0007] Atallah, S. S. , and M. I. Gómez . 2013. Eastern broccoli crop budgets. Charles H. Dyson School of Applied Economics and Management, Cornell University, Ithaca, New York, USA.

[eap2246-bib-0008] Bates D. , Mächler M. , Bolker B. , and Walker S . 2015. Fitting Linear Mixed‐Effects Models Using lme4. Journal of Statistical Software 67 (1). 10.18637/jss.v067.i01.

[eap2246-bib-0009] Benson, J. , R. G. Van Driesche , A. Pasquale , and J. Elkinton . 2003. Introduced braconid parasitoids and range reduction of a native butterfly in New England. Biological Control 28:197–213.

[eap2246-bib-0010] Bianchi, F. J. J. A. , C. J. H. Booij , and T. Tscharntke . 2006. Sustainable pest regulation in agricultural landscapes: A review on landscape composition, biodiversity and natural pest control. Proceedings of the Royal Society B 273:1715–1727. 16790403 10.1098/rspb.2006.3530 PMC1634792

[eap2246-bib-0011] Bronars, S. 2014. No longer home grown: How labor shortages are increasing America’s reliance on imported fresh produce and hampering U.S. economic growth. M. Zeitlin , editor. A New American Economy and The Agriculture Coalition for Immigration Reform.

[eap2246-bib-0012] Burnham, K. P. , and R. Anderson . 2002. Model Selection and multimodel inference: a practical information‐theoretic approach. Springer‐Verlag, New York, New York, USA.

[eap2246-bib-0013] Cantelo, W. W. , and L. L. Sanford . 1984. Insect population response to mixed and uniform plantings of resistant and susceptible plant material. Environmental Entomology 13:1443–1445.

[eap2246-bib-0014] Cartea, M. E. , M. Francisco , M. Lema , P. Soengas , and P. Velasco . 2010. Resistance of Cabbage (*Brassica oleracea* capitata Group) Crops to Mamestra brassicae. Journal of Economic Entomology 103:1866–1874. 21061991 10.1603/ec09375

[eap2246-bib-0015] Casagrande M. , Alletto L. , Naudin C. , Lenoir A. , Siah A. , and Celette F. 2017. Enhancing planned and associated biodiversity in French farming systems. Agronomy for Sustainable Development 37 (6). 10.1007/s13593-017-0463-5.

[eap2246-bib-0016] Chaplin‐Kramer, R. , P. de Valpine , N. J. Mills , and C. Kremen . 2013. Detecting pest control services across spatial and temporal scales. Agriculture, Ecosystems and Environment 181:206–212.

[eap2246-bib-0017] Chaplin‐Kramer, R. , M. E. O’Rourke , E. J. Blitzer , and C. Kremen . 2011. A meta‐analysis of crop pest and natural enemy response to landscape complexity. Ecology Letters 14:922–932. 21707902 10.1111/j.1461-0248.2011.01642.x

[eap2246-bib-0018] Choi, S. , S. Park , Y. P. Lim , S. Kim , J. Park , and G. An . 2014. Metabolite profiles of glucosinolates in cabbage varieties (*Brassica oleracea* var. *capitata*) by season. Color, and Tissue Position 55:237–238.

[eap2246-bib-0019] Crawford, K. M. , and J. A. Rudgers . 2013. Genetic diversity within a dominant plant outweighs plant species diversity in structuring an arthropod community. Ecology 94:1025–1035. 23858643 10.1890/12-1468.1

[eap2246-bib-0020] Crutsinger, G. M. , M. D. Collins , Fordyce, J. A. , J. A. Fordyce , Z. Gompert , C. C. Nice , and N. J. Sanders . 2006. Plant genotypic diversity predicts community structure and governs an ecosystem process. Science 313:966–968. 16917062 10.1126/science.1128326

[eap2246-bib-0021] DeMott, W. R. 1998. Utilization of a *Cyanobacterium* and a phosphorus‐deficient green alga as complementary resources by Daphnids. Ecology 79:2463–2481.

[eap2246-bib-0022] Duvall, V. 2017. Worker shortage threatens U.S. Ag Sustainability. https://www.fb.org/viewpoints/worker‐shortage‐threatens‐u.s.‐ag‐sustainability

[eap2246-bib-0023] Fox, J. , and S. Weisberg . 2011. An R companion to applied regression. Second edition. Second, Thousand Oaks, California, USA.

[eap2246-bib-0024] Gardiner, M. M. , D. A. Landis , C. Gratton , C. D. DiFonzo , M. O’Neal , J. M. Chacon , M. T. Wayo , N. P. Schmidt , E. E. Mueller , and G. E. Heimpel . 2009. Landscape diversity enhances biological control of an introduced crop pest in the North‐Central USA. Ecological Applications 19:143–154. 19323179 10.1890/07-1265.1

[eap2246-bib-0025] Gliessman, S. 1985. Multiple cropping systems: a basis for developing an alternative agriculture. Pages 69–86 *In* Innovative technologies for lesser developed countries, Washington, D.C.: Office of Technology Assessment.

[eap2246-bib-0026] Green, J. P. , R. Foster , L. Wilkins , D. Osorio , and S. E. Hartley . 2015. Leaf colour as a signal of chemical defence to insect herbivores in wild cabbage (*Brassica oleracea*). PLoS ONE 10:1–20. 10.1371/journal.pone.0136884 PMC4564265 26353086

[eap2246-bib-0027] Guerena, M. 2006. Cole Crops and Other Brassicas : ATTRA Organic Production. ATTRA‐National Sustainable Agriculture Information Service. Pages 1–20. Butte, Montana: National Center for Appropriate Technology (NCAT).

[eap2246-bib-0028] Harrison, X. A. 2014. Using observation‐level random effects to model overdispersion in count data in ecology and evolution. PeerJ 2:e616. 25320683 10.7717/peerj.616 PMC4194460

[eap2246-bib-0029] Hartig, F. 2017. DHARMa: Residual Diagnostics for Hierarchical (Multi‐Level/Mixed) Regression Models. R package version 0.1.5. https://cran.r‐project.org/web/packages/DHARMa/vignettes/DHARMa.html

[eap2246-bib-0030] Heath, J. J. , D. F. Cipollini , and J. O. Stireman . 2013. The role of carotenoids and their derivatives in mediating interactions between insects and their environment. Arthropod‐Plant Interactions 7:1–20.

[eap2246-bib-0031] Herlihy, A. M. V. et al 2012. Distribution of *Cotesia rubecula* (Hymenoptera: Braconidae) and its displacement of *Cotesia glomerata* in Eastern North America (Hymenoptera: Braconidae). Florida Entomologist 95:461–467.

[eap2246-bib-0032] Herve, M. 2017. RVAideMemoire: Diverse Basic Statistical and Graphical Functions. R package version 0.9‐66. https://cran.microsoft.com/snapshot/2017‐08‐01/web/packages/RVAideMemoire/index.html

[eap2246-bib-0033] Jones, T. S. , E. Allan , S. A. Härri , J. Krauss , C. B. Müller , and F. J. F. Van Veen . 2011. Effects of genetic diversity of grass on insect species diversity at higher trophic levels are not due to cascading diversity effects. Oikos 120:1031–1036.

[eap2246-bib-0034] Karban, R. , and A. A. Agrawal . 2002. Herbivore offense. Annual Review of Ecology and Systematics 33:641–664.

[eap2246-bib-0035] Karp, D. S. et al 2018. Crop pests and predators exhibit inconsistent responses to surrounding landscape composition. Proceedings of the National Academy of Sciences USA 115:E7863–E7870. 10.1073/pnas.1800042115 PMC6099893 30072434

[eap2246-bib-0036] Koricheva J. , and Hayes D. 2018. The relative importance of plant intraspecific diversity in structuring arthropod communities: A meta‐analysis. Functional Ecology 32 (7):1704 –1717. 10.1111/1365-2435.13062.

[eap2246-bib-0037] Lamers, H. A. H. , F. Kruijssen , B. Sthapit , and R. Rao . 2016. How can markets contribute to conservation of agricultural biodiversity on farms: From theory into practise. Pages 263–284 *in* B. R. Sthapit , H. A. H. Lamers , R. Rao , and A. Bailey , editors. Tropical fruit tree diversity: good practices for in situ and on‐farm conservation. Routledge, Abingdon, UK.

[eap2246-bib-0038] Landis, D. A. , S. D. Wratten , and G. M. Gurr . 2000. Habitat management to conserve natural enemies of arthropod pests in agriculture. Annual Review of Entomology 45:175–201. 10.1146/annurev.ento.45.1.175 10761575

[eap2246-bib-0039] Lenth, R. V. 2016. Least‐squares means: the R package lsmeans. Journal of Statistical Software 69:1–33.

[eap2246-bib-0040] Letourneau, D. K. et al 2011. Does plant diversity benefit agroecosystems? A synthetic review. Ecological Applications 21:9–21. 21516884 10.1890/09-2026.1

[eap2246-bib-0041] Lev‐Yadun, S. , and K. S. Gould . 2009. Role of anthocyanins in plant defence. Pages 22–28 *in* K. S. Gould , K. Davies , and C. Winefield , editors. Anthocyanins: biosynthesis, functions, and applications. Springer, Berlin, Germany.

[eap2246-bib-0042] Macharia, I. , B. Lo , and H. De Groote . 2005. Assessing the potential impact of biological control of Plutella xylostella (diamondback moth) in cabbage production in Kenya. Crop Protection 24:981–989.

[eap2246-bib-0043] Malenčić, D. , J. Cvejić , and J. Miladinović . 2012. Polyphenol content and antioxidant properties of colored soybean seeds from central Europe. Journal of Medicinal Food 15:89–95. 21861721 10.1089/jmf.2010.0329 PMC3249631

[eap2246-bib-0044] McArt, S. H. , and J. S. Thaler 2013. Plant genotypic diversity reduces the rate of consumer resource utilization. Proceedings of the Royal Society B 280:20130639. 23658201 10.1098/rspb.2013.0639 PMC3673052

[eap2246-bib-0045] McCarl, B. A. 1982. Cropping activities in agricultural sector models: a methodological proposal. American Journal of Agricultural Economics 64:768–772.

[eap2246-bib-0046] Meehan, T. D. , B. P. Werling , D. A. Landis , and C. Gratton . 2011. Agricultural landscape simplification and insecticide use in the Midwestern United States. Proceedings of the National Academy of Sciences USA 108:11500–11505. 10.1073/pnas.1100751108 PMC3136260 21746934

[eap2246-bib-0047] Mundt, C. C. 2002. Use of multiline cultivars and cultivar mixtures for disease management. Annual Review of Phytopathology 40:381–410. 10.1146/annurev.phyto.40.011402.113723 12147765

[eap2246-bib-0048] Nielsen, J. K. 1988. Crucifer‐feeding Chrysomelidae: Mechanisms of host plant finding and acceptance. *In* P. Jolivet , E. Petipierre , and T. H. Hsiao , editors. Biology of Chrysomelidae. Dordrecht, The Netherlands.

[eap2246-bib-0049] Park, S.‐Y. , S.‐H. Lim , S.‐H. Ha , Y. Yeo , W. T. Park , D. Y. Kwon , S. U. Park , and J. K. Kim . 2013. Metabolite profiling approach reveals the interface of primary and secondary metabolism in colored cauliflowers (*Brassica oleracea* L. ssp. *botrytis*). Journal of Agricultural and Food Chemistry 61:6999–7007. 23782237 10.1021/jf401330e

[eap2246-bib-0050] Power, A. G. 1988. Leafhopper response to genetically diverse maize stands. Entomologia Experimentalis et Applicata 49:213–219.

[eap2246-bib-0100] Power, A. G. 1991. Virus spread and vector dynamics in genetically diverse plant populations. Ecology. 72:232–241.

[eap2246-bib-0051] Power, A. G. 2010. Ecosystem services and agriculture: tradeoffs and synergies. Philosophical Transactions of the Royal Society B 365:2959–2971. 10.1098/rstb.2010.0143 PMC2935121 20713396

[eap2246-bib-0052] R Core Team 2017. R: A language and environment for statistical computing. R Foundation for Statistical Computing, Vienna, Austria. www.R‐project.org

[eap2246-bib-0053] Reiss, E. R. , and L. E. Drinkwater . 2018. Cultivar mixtures: A meta‐analysis of the effect of intraspecific diversity on crop yield: A. Ecological Applications 28:62–77. 28940830 10.1002/eap.1629

[eap2246-bib-0054] Renwick, J. A. A. , and C. Radke . 1988. Sensory cues in host selection of oviposition by the cabbage butterfly, Pieris rapae. Journal of Insect Ecology 34:251–257.

[eap2246-bib-0055] Root, R. B. 1973. Organization of a plant‐arthropod association in simple and diverse habitats: the fauna of collards (*Brassica oleracea*). Ecological Monographs 43:95–124.

[eap2246-bib-0056] Santolamazza‐Carbone, S. , P. Velasco , J. Selfa , P. Soengas , and M. E. Cartea . 2013. Intraspecific variation of host plant and locality influence the Lepidopteran‐parasitoid system of Brassica oleracea crops. Journal of Economic Entomology 106:1134–1144. 23865177 10.1603/ec12481

[eap2246-bib-0057] Schuler, M. A. 2011. P450s in plant‐insect interactions. Biochimica et Biophysica Acta 1814:36–45. 20883828 10.1016/j.bbapap.2010.09.012

[eap2246-bib-0058] Seaman, A. 2013. 2013 organic production guide for cole crops. Cornell University, Ithaca, New York, USA.

[eap2246-bib-0059] Seaman, A. , editor. 2016. Production guide for organic cole crops: cabbage, cauliflower, broccoli, and brussels sprouts. New York State Integrated Pest Management Program, Cornell University (New York State Agricultural Experiment Station), Geneva, New York, USA.

[eap2246-bib-0060] Shelton, A. M. , W. T. Wilsey , E. R. Hoebeke , and M. A. Schmaedick . 2002. Parasitoids of cabbage lepidopteran in central New York. Journal of Entomological Science 37:270–271.

[eap2246-bib-0061] Sinkkonen, A. , E. Somerkoski , U. Paaso , J. K. Holopainen , M. Rousi , and J. Mikola . 2012. Genotypic variation in yellow autumn leaf colours explains aphid load in silver birch. New Phytologist 195:461–469. 10.1111/j.1469-8137.2012.04156.x 22548444

[eap2246-bib-0062] Snyder, L. , M. Gomez , E. Mudrak , and A. Power . 2020. Data from: Landscape‐dependent effects of varietal mixtures on insect pest control and implications for farmer profits. Dryad, data set. 10.5061/dryad.0gb5mkkzc PMC7988554 33124091

[eap2246-bib-0063] Strauch, R. C. , M. F. Mengist , K. Pan , G. G. Yousef , M. Iorizzo , A. F. Brown , and M. A. Lila . 2019. Variation in anthocyanin profiles of 27 genotypes of red cabbage over two growing seasons. Food Chemistry 301:125289. 31387047 10.1016/j.foodchem.2019.125289

[eap2246-bib-0064] Tilman, D. 1982. Resource competition and community structure. Princeton University Press, Princeton, New Jersey, USA. 7162524

[eap2246-bib-0065] Tooker, J. F. , and S. D. Frank . 2012. Genotypically diverse cultivar mixtures for insect pest management and increased crop yields. Journal of Applied Ecology 49:974–985.

[eap2246-bib-0066] Tscharntke, T. et al 2012. Landscape moderation of biodiversity patterns and processes—eight hypotheses. Biological Reviews 87:661–685. 22272640 10.1111/j.1469-185X.2011.00216.x

[eap2246-bib-0067] Tscharntke, T. , R. Bommarco , Y. Clough , T. O. Crist , D. Kleijn , T. A. Rand , J. M. Tylianakis , S. van Nouhuys , and S. Vidal . 2007. Conservation biological control and enemy diversity on a landscape scale. Biological Control 43:294–309.

[eap2246-bib-0068] Tsuji, J. , and L. Coe . 2014. Effects of foliage color on the landing response of *Pieris rapae* (Lepidoptera: Pieridae). Environmental Entomology 43:989–994. 25182617 10.1603/EN14084

[eap2246-bib-0069] Veres, A. , S. Petit , C. Conord , and C. Lavigne . 2013. Does landscape composition affect pest abundance and their control by natural enemies? A review. Agriculture, Ecosystems and Environment 166:110–117.

[eap2246-bib-0070] Wetzel, W. C. , N. C. Aflitto , and J. S. Thaler . 2018. Plant genotypic diversity interacts with predation risk to influence and insect herbivore across its ontogeny. Ecology 99:2338–2347. 30047598 10.1002/ecy.2472

[eap2246-bib-0071] Wetzel, W. C. , H. M. Kharouba , M. Robinson , M. Holyoak , and R. Karban . 2016. Variability in plant nutrients reduces insect herbivore performance. Nature 539:425–427. 27749815 10.1038/nature20140

[eap2246-bib-0072] Wetzel, W. C. , and J. S. Thaler . 2016. Does plant trait diversity reduce the ability of herbivores to defend against predators? the plant variability‐gut acclimation hypothesis. Current Opinion in Insect Science 14:25–31. 27436643 10.1016/j.cois.2016.01.001

[eap2246-bib-0073] Wiczkowski, W. , J. Topolska , and J. Honke . 2014. Anthocyanins profile and antioxidant capacity of red cabbages are influenced by genotype and vegetation period. Journal of Functional Foods 7:201–211.

[eap2246-bib-0074] Wiswall, R. 2009. *In* B. Watson and L. Jorstad , editors. The organic farmer’s business handbook, White River Junction, VT: Chelsea Green Publishing.

[eap2246-bib-0075] Wood, S. A. , D. S. Karp , F. DeClerck , C. Kremen , S. Naeem , and C. A. Palm . 2015. Functional traits in agriculture: Agrobiodiversity and ecosystem services. Trends in Ecology and Evolution 30:531–539. 26190137 10.1016/j.tree.2015.06.013

[eap2246-bib-0076] Yang E.‐C. , Lee D.‐W. , and Wu W.‐Y. 2003. Action spectra of phototactic responses of the flea beetle, Phyllotreta striolata. Physiological Entomology 28 (4):362 –368. 10.1111/j.1365-3032.2003.00351.x.

[eap2246-bib-0077] Zuur, A. F. , E. N. Ieno , N. J. Walker , A. A. Saveliev , and G. M. Smith . 2009. Mixed effects models and extensions in ecology with R. Pages 1–574 *in* M. Gail , K. Krickeberg , J. Samet , A. Tsiastis , and W. Wong , editors. Mixed effects models and extensions in ecology with R. Springer, New York, New York, USA.

