# Supplementary material for: Landscape‐dependent effects of varietal mixtures on insect pest control and implications for farmer profits
Source: Ecol Appl. 2021 Jan 6;31(2):e02246. doi: 10.1002/eap.2246 (PMC7988554; doi:10.1002/eap.2246)
Supplement: Supplementary file 1 — Appendix S1 [file EAP-31-e02246-s001.pdf]

Supporting Information. Snyder, L.D., M.I. Gómez, E.L. Mudrak, and A.G. Power. 2020. Landscape-dependent effects of varietal mixtures on insect pest control and implications for farmer profits. Ecological Applications.

Appendix S1

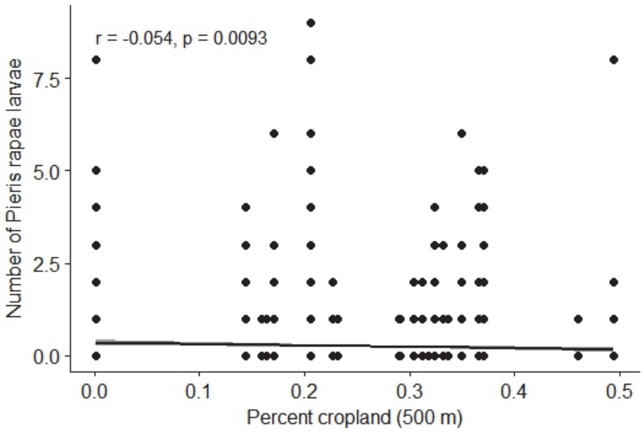

Figure S1. The relationship between percent cropland (500 m) and the abundance of *P. rapae* larvae.

Table S1. List of inputs included in the crop budget analysis. These inputs are representative of those used across all farms included in the study.

**Input Categories**		
**Fertility**	**Pest**	**Weed**
Blood meal<br>Chicken manure<br>Feather meal<br>Foliar kelp extract<br>Green sand<br>Organic compost<br>Organic fish emulsion<br>Organic phosphorus fertilizer	Entrust<br>Protectnet<br>Pyganic<br>Row cover	Hay<br>Straw bale mulch<br>Weed cloth<br>Wood chips

Table S2. Description of crop damage scoring. Methods based on Macharia et al. (2005).

Crop damage score	Damage description
0	No damage, or minor leaf damage restricted to a few, small partial holes (leaf tissue is not fully punctured) on the non-harvested portion of the crop
1	A few, small holes restricted to the non-harvested portion of the crop
2	Considerable damage to the outer and older leaves, slight damage to the harvested portion of the crop
3	Considerable damage to the outer and older leaves, moderate damage to the harvested portion of the crop
4	Considerable damage to the entire plant, but a portion of plant could still be marketable after damaged areas are removed
5	Severe plant damage that renders the crop unmarketable

Table S3. Test of spatial autocorrelation of percent cropland at farm sites within three spatial scales (500, 1000, and 1500 m) in the early and late season. Shown are the results of Mantel tests that analyze the relationship between the Euclidean distance of sites—based upon latitude and longitude—and the difference in percent cropland at each spatial scale.

CROPLAND			
**Season**	**Scale (m)**	**r**	**Significance**
Early Season	500	0.1	0.16
Early Season	1000	0.14	0.14
Early Season	1500	0.14	0.18
Late Season	500	0.11	0.85
Late Season	1000	0.11	0.7
Late Season	1500	0.07	0.45

Table S4. Results of Mantel tests to evaluate spatial autocorrelation between farm fields and response variables in the early and late season.

**Season**	**Response Variable**	**r**	**Significance**
Early Season	Flea beetle abundance	0.009	0.33
Early Season	Crop Damage	0.21	0.08
Late Season	*P. rapae* larval abundance	-0.09	0.88
Late Season	Crop Damage	0.03	0.29

Table S5. Effect of local-scale predictors (varietal richness and color richness), percent cropland, and the two-way interactions between the local-scale predictors and percent cropland on the incidence and abundance of flea beetles and *P. rapae*. Results from the Binomial model (incidence) and the Poisson model (abundance) evaluating the effect of intraspecific crop diversity and landscape composition on pest incidence and abundance. Bold type highlights significant p-values and lowest AIC values, indicating the most predictive spatial scale.

			VARIETAL RICHNESS		COLOR RICHNESS		PERCENT CROPLAND		VARIETAL RICHNESS X CROPLAND		COLOR RICHNESS X CROPLAND	
**Response Variable**	**Explanatory Scale (m)**	**Model AIC**	**estimate**	**P**	**estimate**	**P**	**estimate**	**P**	**estimate**	**P**	**estimate**	**P**
Early Season Flea Beetle Incidence	500	1272.5	3.450	**0.035**	0.184	0.953	20.094	0.167	-9.902	**0.020**	-1.803	0.846
	1000	**1269.7**	4.642	**0.011**	-1.588	0.642	18.795	0.121	-12.845	**0.007**	3.109	0.743
	1500	NA	NA	NA	NA	NA	NA	NA	NA	NA	NA	NA
Early Season Flea Beetle Abundance	500	5308.8	0.551	0.268	-0.326	0.770	0.749	0.895	-1.493	0.247	0.885	0.798
	1000	**5307.4**	0.854	0.134	-0.782	0.488	0.228	0.956	-2.259	0.129	2.111	0.503
	1500	5308.1	0.791	0.193	-0.665	0.561	0.983	0.818	-2.016	0.187	1.551	0.625
Late Season *P. rapae* Incidence	500	**1306.6**	-2.543	**0.002**	-0.222	0.921	-21.126	0.133	6.993	**0.004**	-0.802	0.915
	1000	1310.9	-1.724	**0.027**	-0.112	0.949	-11.403	0.273	4.459	0.055	-2.111	0.691
	1500	1312.7	-1.362	0.079	0.196	0.907	-5.442	0.611	3.513	0.151	-3.304	0.555
Late Season *P. rapae* Abundance	500	**954.2**	0.087	0.646	-1.353	**0.001**	-6.687	**0.015**	-0.283	0.618	3.724	**0.009**
	1000	956.5	0.043	0.834	-1.000	**0.023**	-4.489	0.088	-0.079	0.893	2.165	0.112
	1500	957.9	0.115	0.59	-0.828	0.056	-2.968	0.249	-0.325	0.616	1.814	0.209

Table S6. Effect of varietal richness and color richness on profitability, revenue, labor costs, and input costs. Results from linear mixed effects models evaluating the potential for intraspecific crop diversity to support economic services for growers. Bold type shows significant p-values.

	VARIETAL RICHNESS		COLOR RICHNESS		
**Response variable**	**Estimate**	**Uncorrected p values**	**Estimate**	**Uncorrected p values**	**Corrected p values**
Profitability	-0.580	0.421	1.848	**0.014**	**0.042**
Revenue	0.064	0.855	0.289	**0.002**	**0.008**
Labor Cost	-0.014	0.542	-0.034	**0.033**	0.066
Input Cost	-0.471	0.312	-0.457	0.068	0.068

LITERATURE CITED

Macharia, I., B. Lo, and H. De Groote. 2005. Assessing the potential impact of biological control of *Plutella xylostella* (diamondback moth) in cabbage production in Kenya. Crop Protection 24:981–989.
